# Clinical Outcomes of Cetuximab and Paclitaxel after Progression on Immune Checkpoint Inhibitors in Recurrent or Metastatic Head and Neck Squamous Cell Carcinoma

**DOI:** 10.3390/medicina57111151

**Published:** 2021-10-23

**Authors:** Shinsuke Suzuki, Satoshi Toyoma, Yohei Kawasaki, Koh Koizumi, Nobuko Iikawa, Kazuhiro Shiina, Tentaro Endo, Tomoe Abe, Teppei Kouga, Takechiyo Yamada

**Affiliations:** Department of Otorhinolaryngology & Head and Neck Surgery, Graduate School of Medicine, Akita University, Akita 010-8543, Japan; s.toyoma@med.akita-u.ac.jp (S.T.); kawa0807@med.akita-u.ac.jp (Y.K.); kkoizumi@med.akita-u.ac.jp (K.K.); iikawan@med.akita-u.ac.jp (N.I.); k.shiina@med.akita-u.ac.jp (K.S.); t.endo.oto@med.akita-u.ac.jp (T.E.); emotobea@med.akita-u.ac.jp (T.A.); t.kogaaaa@med.akita-u.ac.jp (T.K.); ymdtkcy@ned.akita-u.ac.jp (T.Y.)

**Keywords:** head and neck squamous cell carcinoma, cetuximab, paclitaxel, immune checkpoint inhibitor, chemotherapy

## Abstract

*Background and Objectives*: In recent years, the effectiveness of chemotherapy after immune checkpoint inhibitor administration has attracted attention in various cancers, including head and neck cancers. However, individual assessments of the administered chemotherapy regimens are insufficient. This study aimed to evaluate the efficacy and safety of chemotherapy after immune checkpoint inhibitor administration in recurrent metastatic head and neck cancer by focusing on a single regimen. *Materials and Methods*: We retrospectively reviewed clinical and radiological data from the medical records of 18 patients with recurrent or metastatic (R/M) head and neck squamous cell carcinoma (HNSCC) who received systemic chemotherapy with weekly cetuximab and paclitaxel (Cmab + PTX) after progression following immune checkpoint inhibitor (ICI) therapy. The objective response rate (ORR) and disease control rate (DCR) were assessed using Response Evaluation Criteria in Solid Tumors (RECIST) version 1.1. Progression-free survival (PFS) and overall survival (OS) were estimated using the Kaplan–Meier method. Adverse events (AEs) were recorded using National Cancer Institute Common Terminology Criteria for Adverse Events, version 4.0. *Results*: In all patients, the ORR, DCR, median PFS, and median OS were 44.4%, 72.2%, 3.8 months, and 9.6 months, respectively. Regarding AEs, three patients developed grade 3 neutropenia. Grade 3 anemia, paronychia, asthenia, and peripheral neuropathy were observed in one patient each. There were no treatment-related deaths. *Conclusions*: Cmab + PTX was shown to maintain high efficacy and acceptable safety for R/M HNSCC that progressed after ICI therapy. Further research is needed to establish optimal treatment sequences and drug combinations for recurrent R/M HNSCC.

## 1. Introduction

During the treatment of head and neck squamous cell carcinoma (HNSCC), recurrence and metastasis are common and contribute to poor prognosis [1,2]. Therefore, improving the prognosis of recurrent or metastatic (R/M) cases remains important for the treatment of HNSCC. Various systemic and local treatments have been evaluated to improve patient outcomes, particularly drug therapy, which is the primary treatment for R/M HNSCC [3].

For many years, drug therapy for R/M HNSCC has been centered around cytotoxic drugs, such as cisplatin; however, there is currently no consensus on treatment or treatment sequence. In recent years, epidermal growth factor receptor (EGFR) inhibitors and immune checkpoint inhibitors (ICIs) have been introduced, establishing a standard first-line treatment for R/M HNSCC [1,4].

Recently, conventional cytotoxic chemotherapy administered after ICI therapy failure has been reported to be highly effective in some solid tumors, including lung and gastric cancer [5,6]. Reports of the efficacy of chemotherapy after progression following ICI therapy in head and neck cancer are also accumulating [7,8,9], which remains important for considering the treatment sequence in R/M HNSCC. Therefore, it is necessary to elucidate the mechanism whereby chemotherapies are highly effective after ICI therapy and identify the most effective chemotherapy option after progression following ICI therapy in R/M HNSCC.

Combination therapy with cetuximab and paclitaxel (Cmab + PTX) is a regimen for R/M HNSCC that is highly effective as first-line treatment [10,11,12]. Additionally, Cmab + PTX has recently been reported to be effective when administered after ICI therapy failure. However, Cmab + PTX is only one of several regimens that have been evaluated, and its efficacy and safety have not been thoroughly studied [8,9,13].

The purpose of the present study was to evaluate the efficacy and safety of Cmab + PTX in progressive disease following ICI therapy.

## 2. Materials and Methods

### 2.1. Patients and Data Collection

We retrospectively reviewed clinical and radiological data from the medical records of patients with R/M HNSCC who received systemic chemotherapy, weekly cetuximab (400 mg/m^2^ (load), 250 mg/m^2^ (maintenance)) plus paclitaxel (80 mg/m^2^) after progression following ICI therapy between October 2017 and December 2020 at Akita University Hospital. Patients with an Eastern Cooperative Oncology Group (ECOG) performance status of 0–2 and adequate laboratory status were included in this study. The patients were followed up until death or the cutoff date (1 July 2021).

The study was approved by the Institutional Review Board of Akita University Hospital and was conducted in accordance with the principles of the Declaration of Helsinki. The requirement for obtaining informed consent was waived as the study was a retrospective analysis of existing administrative and clinical data.

Tumor response was assessed according to the Response Evaluation Criteria in Solid Tumors (RECIST), version 1.1. The objective response rate (ORR) was defined as the proportion of patients who exhibited complete response (CR) or partial response (PR) as the best response. The disease control rate (DCR) was defined as the proportion of patients who exhibited CR, PR, or stable disease (SD) as the best response. The clinical response to treatment was evaluated using computed tomography every 8–12 weeks.

Progression-free survival (PFS) was defined as the time from the initiation of systemic chemotherapy after progression following ICI therapy until disease progression, death due to any cause, or the cutoff date when no progression was observed. Overall survival (OS) was defined as the time from the initiation of systemic chemotherapy after progression following ICI therapy until death due to any cause or the cutoff date. Adverse events (AEs) were recorded using the National Cancer Institute Common Terminology Criteria for Adverse Events, version 4.0.

### 2.2. Statistical Analysis

Survival curves were estimated using the Kaplan–Meier method. The ORR associated with Cmab + PTX and its 95% confidence interval (CI) were calculated. The association between ORR and patient characteristics was tested using the chi-squared test or Fisher’s exact test for comparing two groups according to the sample size, and the Kruskal–Wallis test was performed for comparisons between three or more groups. Statistical analyses were performed using EZR version 4.0.0 (Saitama Medical Center, Jichi Medical University, Saitama, Japan). *p* values of <0.05 were considered statistically significant.

## 3. Results

### 3.1. Patient Characteristics

Eighteen patients (17 males and 1 female) were eligible for this study, and their characteristics are summarized in Table 1. The median age was 61.5 years (range, 40–78 years). Most patients had an ECOG performance status of 0 or 1, but two patients had a PS of 2. The hypopharynx was the most common primary site in seven cases, followed by the oral cavity in five cases and the oropharynx in three cases. The lesion sites evaluated were locoregional in 14 cases and distant metastases in 4 cases. Chemotherapy was provided for eight patients before ICI therapy, and all of them used regimens that included cetuximab. In six of these patients, cetuximab + cisplatin + fluorouracil was administered, and cetuximab + paclitaxel + carboplatin was administered in two patients. In the remaining 10 cases, ICIs were administered as the initial treatment. The ICI administered was nivolumab in 14 cases and pembrolizumab in 4 cases. The median follow-up period was 7.7 months, and 9 (50.0%) patients died during the study period.

### 3.2. Efficacy of Chemotherapy after ICI Therapy

**Figure 1:**
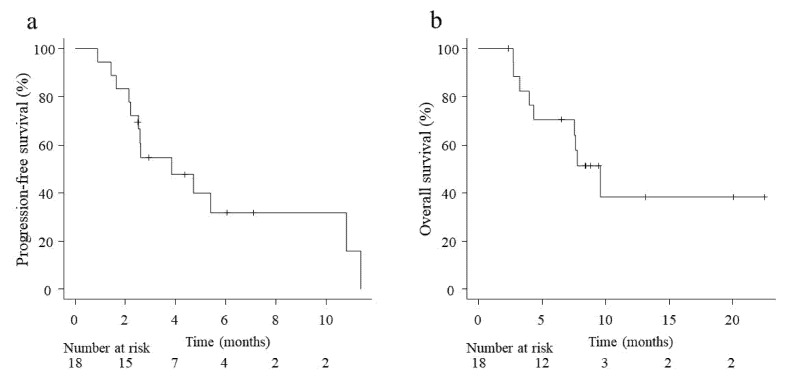
Kaplan–Meier curve of progression-free survival (**a**) and overall survival (**b**) of patients on cetuximab and paclitaxel chemotherapy after progression following immune checkpoint inhibitor therapy.

Among the total population, two (11.1%) patients achieved CR, six (33.3%) achieved PR, five (27.8%) achieved SD, and five (27.8%) achieved progressive disease as their best response on Cmab + PTX after ICI therapy, yielding an ORR and DCR of 44.4% (8 of 18 patients, 95% CI, 21–69.2%) and 72.2% (13 of 18 patients, 95% CI, 46.5–90.3%), respectively. The median PFS and OS were 3.8 (95% CI, 2.2–10.8) and 9.6 (95% CI, 4.0–not estimable) months, respectively (Figure 1).

We analyzed the prognostic factors of ORR after Cmab + PTX after progression following ICI therapy. The patients’ sex, age, PS, initial tumor location, site of progression after ICI therapy, type of ICI, and best response to ICI therapy were not associated with the response to Cmab + PTX after ICI therapy. Additionally, there were no significant differences in ORR between patients with or without a history of cetuximab-containing chemotherapy prior to ICI therapy (Table 2).

### 3.3. Safety

AEs observed during Cmab + PTX chemotherapy after progression following ICI therapy are listed in Table 3. The most common AEs of all grades were acneiform rash, neutropenia, and anemia in 15, 14, and 13 patients, respectively. Three patients (16.7%) developed grade 3 neutropenia. Grade 3 anemia, paronychia, asthenia, and peripheral neuropathy were observed in one patient each (Table 3). Three patients who developed grade 3 neutropenia and one patient with prolonged grade 2 asthenia underwent paclitaxel withdrawal and received cetuximab maintenance monotherapy. There were no treatment-related deaths.

## 4. Discussion

Although pharmacotherapy is essential for the treatment of R/M HNSCC, a consensus standard treatment for R/M HNSCC has not been defined. Therefore, various drugs have been used to enhance the therapeutic effects, and the timing and combination of these drugs have also been investigated [4].

The EXTREME regimen with the addition of the EGFR inhibitor cetuximab to platinum and fluorouracil resulted in a significant prolongation of OS in patients with R/M HNSCC [14]. This was a major finding in the treatment of R/M HNSCC, for which no standard treatment has existed. Subsequently, ICI therapy has shown significant promise [15,16], and R/M HNSCC treatment is now entering a new era with a focus on tumor immunity.

However, the advent of ICI does not mean that conventional cytotoxic chemotherapeutic agents are no longer effective. Currently, the PD-1 inhibitors nivolumab and pembrolizumab are used as ICIs in head and neck cancers. However, in their respective phase III studies, nivolumab was evaluated in platinum-resistant R/M HNSCC [15], whereas pembrolizumab was studied in platinum-naive R/M HNSCC [16]. Platinum remains a cornerstone cytotoxic chemotherapeutic agent for HNSCC. Differences in the history of platinum use under conditions in which each ICI was found to be effective should be noted. Additionally, to maximize the effect of pembrolizumab in R/M HNSCC, it is necessary to combine it with standard chemotherapy depending on PD-L1 expression levels in the tumor [16].

The combination of ICI therapy and chemotherapy has a synergistic effect, and recent studies have confirmed its efficacy [17]. Chemotherapy has also been shown to be more effective in R/M HNSCC after progression following ICI therapy [8,9,18]. This emphasizes the importance of not only the use of ICI and chemotherapy in combination but also the treatment sequence of ICI and chemotherapy. Therefore, with the advent of ICI therapy, the importance of chemotherapy has increased. To determine the most effective treatment for R/M HNSCC, it is necessary to have a deeper understanding of the underlying mechanism of ICIs and chemotherapy combined with ICIs.

The reason for the high efficacy of Cmab + PTX is that cetuximab enhances the effect of PTX by downregulating p65 expression induced by paclitaxel [19]. Cmab + PTX may be used in patients for whom platinum is not recommended or when platinum resistance develops. Furthermore, because it shows a high response rate, Cmab + PTX may be administered not only as first-line treatment for R/M HNSCC [11,12] but also as second- and third-line treatment. In fact, it is one of the most effective regimens after ICI therapy according to many reports [8,9,13] and is considered the primary treatment option for R/M HNSCC.

Cetuximab used in the Cmab + PTX regimen is known to induce antibody-dependent cellular cytotoxicity (ADCC) as well as direct cytotoxicity through EGFR inhibition [20,21]. Because ICIs were found to enhance cetuximab-induced ADCC [20], cetuximab and ICI are expected to have a synergistic antitumor effect through ADCC. In fact, the combination of cetuximab and the PD-1 inhibitor pembrolizumab showed promising activity in patients with R/M HNSCC [22].

The efficacy of cetuximab-containing regimens after the use of ICIs in R/M HNSCC has been recently reported, and this may be the result of a synergistic effect of ICIs and cetuximab, as described above. However, multiple regimens including cetuximab have been evaluated in these reports, and individual regimens were not considered [18,23]. This is the first report to describe the efficacy and safety of a regimen limited to Cmab + PTX after progression following ICI therapy, which has important implications for the optimization of the treatment sequence for R/M HNSCC.

In the present study, the ORR was 44.4% and DCR was 72.2%. In previous studies in which Cmab + PTX was administered as first-line therapy for R/M HNSCC, ORR and DCR were generally comparable, i.e., in the range of 46–52% and 73–83%, respectively [10,11,12]. It should be noted that the ORR did not decrease with prior cetuximab administration in this study. Kurosaki et al. reported that cetuximab-containing chemotherapy administered after ICI therapy tended to result in better ORR and OS in patients with R/M HNSCC who had previously received cetuximab than that in patients who had not [23]. The authors cited a study of metastatic colorectal cancer as the reason for this effect. In this study, anti-EGFR-treatment-resistant clones produced by cetuximab were thought to have been reduced by subsequent chemotherapy without anti-EGFR antibodies [24]. The authors speculated that ICIs may have circumvented the resistance to cetuximab. In the present study, there was no significant enhancement of the effect of post-ICI Cmab + PTX in patients with a history of cetuximab administration; however, we believe that the significance of cetuximab re-administration should be further investigated.

With respect to safety, in a study of Cmab + PTX administered as first-line therapy in R/M HNSCC, the major AEs were acne, anemia, and neutropenia, and the reported frequencies of grade 3 and higher AEs were 0–24%, 13–17%, and 13–15%, respectively [10,11,12]. Among the grade 3 or higher AEs observed in our study, neutropenia had the highest incidence (three cases; 16.7%); however, no patient had grade 3 or higher acne or anemia. Collectively, these results indicate the effectiveness of Cmab + PTX even after ICI therapy without any increase in risk. This is a significant advantage when considering the therapeutic sequence for R/M HNSCC.

The median PFS and OS obtained in the present study were 3.8 and 9.6 months, respectively. In similar published reports, they were 4.2–7.0 and 8.1–16.3 months, respectively [10,11,12]. Thus, the PFS in our study tended to be slightly shorter. The reason for this may be that the median observation period was 7.7 months, which is insufficient to evaluate survival. In fact, of the 18 patients included in this study, 5 remained on Cmab + PTX with SD or better at the cutoff date, whereas 9 were survivors. Continued long-term observation will be necessary for a detailed analysis of PFS and OS.

The limitations to the present study include the short observation period mentioned above and the fact that it was a single-center retrospective study with a relatively small number of patients. To optimize R/M HNSCC pharmacotherapy, further individual studies on chemotherapy after ICI are needed.

## 5. Conclusions

Our findings indicate that Cmab + PTX is an effective and well-tolerated treatment even after progression following ICI therapy. Additionally, the effect of Cmab + PTX was not attenuated by cetuximab administration before ICI therapy. Our results suggest that Cmab + PTX should be actively considered for progressive disease after ICI treatment of R/M HNSCC. Further studies and clinical trials will be necessary to confirm these results.

## Figures and Tables

**Table 1:** Characteristics of the patients (*N* = 18).

Characteristic	No. of Patients	Percent
Age (years), median (range)	61.5 (40–78)	
**Sex**		
Male	17	94.4
Female	1	5.6
**EOCG performance status**		
0	6	33.3
1	10	55.6
2	2	11.1
**Initial tumor location**		
Oral	5	27.8
Oropharynx	3	16.7
Hypopharynx	7	38.9
Others	3	16.7
**Site of progression after ICI therapy**		
Locoregional	14	77.8
Distant	4	22.2
**ICI**		
Nivolumab	14	77.8
Pembrolizumab	4	22.2
**Cetuximab before ICI therapy**		
Yes	8	44.4
No	10	55.6

Abbreviations: EOCG, Eastern Cooperative Oncology Group; ICI, immune checkpoint inhibitor.

**Table 2:** Prognostic analysis of ORR after chemotherapy following ICI therapy.

Patients	CR-PR	SD-PD	ORR (%)	*p* Value
**Sex**				
Male	8	9	47.1	0.36
Female	0	1	0.0	
**Age (years)**				
>60	4	6	40.0	0.67
<60	4	4	50.0	
**Performance status**				
0.1	7	9	43.8	0.87
2	1	1	50.0	
**Initial tumor location**				
Hypopharynx	2	5	28.6	0.71
Oropharynx	2	1	66.7	
Oral	3	2	60.0	
Nasal	1	2	33.3	
**Site of progression after ICI therapy**				
Locoregional	7	7	50.0	0.38
Distant	1	3	25.0	
**Type of ICI**				
Nivo	7	7	50.0	0.38
Pemb	1	3	25.0	
**Best response to ICI**				
CR, PR	3	2	60.0	0.41
SD, PD	5	8	38.5	
**Cetuximab-containing chemotherapy before ICI therapy**				
Yes	2	5	28.6	0.28
No	6	5	54.5	

Abbreviations: ORR, objective response rate; CR, complete response; PR, partial response; SD, stabel disease; PD, progression disease; ICI, immune checkpoint inhibitor.

**Table 3:** Toxicity.

	All Grades	Grade 3
	Patients, *n* (%)	Patients, *n* (%)
Hematologic		
Neutropenia	14 (77.8)	3 (16.7)
Anemia	13 (72.2)	1 (5.6)
Thrombocytopenia	9 (50.0)	0
Non-hematologic		
Hypomagnesemia	4 (22.2)	0
Acne-like rash	15 (83.3)	0
Paronychia	3 (16.7)	1 (5.6)
Asthenia	1 (5.6)	1 (5.6)
Peripheral neuropathy	3 (16.7)	1 (5.6)

## Data Availability

The datasets used and analyzed during the current study are available from the corresponding author on reasonable request.
